# A Review of *In Vitro* and *In Vivo* Studies on the Efficacy of Herbal Medicines for Primary Dysmenorrhea

**DOI:** 10.1155/2014/296860

**Published:** 2014-11-05

**Authors:** Kyoung-Sun Park, Kang-In Park, Deok-Sang Hwang, Jin-Moo Lee, Jun-Bock Jang, Chang-Hoon Lee

**Affiliations:** Department of Korean Medicine Obstetrics & Gynecology, College of Korean Medicine, Kyung Hee University, 26 Kyungheedae-ro, Dongdaemun-gu, Seoul 130-701, Republic of Korea

## Abstract

*Purpose*. Primary dysmenorrhea (PD) is a common gynecological complaint among adolescent girls and women of reproductive age. This study aims to review the findings of published articles on the *in vitro* and *in vivo* efficacy of herbal medicines for PD. *Methods*. *In vitro* and *in vivo* studies of herbal compounds, individual herbal extracts, or herbal formula decoctions published from their inception to April 2014 were included in this review. *Results*. A total of 18 studies involving herbal medicines exhibited their inhibitory effect on PD. The majority of *in vitro* studies investigated the inhibition of uterine contractions.* In vivo* studies suggest that herbal medicines exert a peripheral analgesic effect and a possible anti-inflammatory activity via the inhibition of prostaglandin (PG) synthesis. The mechanisms of herbal medicines for PD are associated with PG level reduction, suppression of cyclooxygenase-2 expression, superoxide dismutase activation and malondialdehyde reduction, nitric oxide, inducible nitric oxide synthase, and nuclear factor-kappa B reduction, stimulation of somatostatin receptor, intracellular Ca^2+^ reduction, and recovery of phospholipid metabolism. *Conclusions*. Herbal medicines are thought to be promising sources for the development of effective therapeutic agents for PD. Further investigations on the appropriate herbal formula and their constituents are recommended.

## 1. Introduction

Dysmenorrhea refers to the occurrence of painful cramps in the lower abdominal region during menstruation and is a common gynecological complaint among adolescent girls and women of reproductive age. It is usually classified into two subcategories: primary dysmenorrhea (PD) and secondary dysmenorrhea. PD occurs in the absence of an identifiable pathological condition [1]. When the menstrual pain is associated with an organic pathology such as endometriosis, it is defined as secondary dysmenorrhea [2]. PD characteristically begins at or shortly after menarche, which coincides with the occurrence of the regular ovulatory cycle. Pain usually develops within hours of the start of menstrual bleeding and peaks as the flow becomes the heaviest during the first or second day of the cycle [2]. The prevalence of PD is estimated to be 20 to 90% among women of reproductive age [3, 4], and 15% of female adolescents experience severe PD [5].

Principal pharmacological therapies for PD include nonsteroidal anti-inflammatory drugs (NSAIDs) or oral contraceptive pills (OCPs). NSAIDs reduce myometrial activity by inhibiting prostaglandin (PG) synthesis and reducing vasopressin secretion. However, the failure rate of NSAIDs is often 20 to 25% [6], and these may be contraindicated and not tolerated by some women [7]. Besides, NSAIDs have long-term adverse effects involving disorders of the liver, kidney, and digestive systems [8, 9]. OCPs suppress ovulation and thin the endometrial lining which reduces menstrual fluid volume along with the amount of PG produced, thus reducing the pain associated with uterine contractions [10]. While OCPs can be an effective treatment for PD, they can cause side effects including nausea and water retention and may not be suitable for all women, especially those pursuing pregnancy [11, 12]. Surgical interruption of the pelvic nerve pathways can be used in women who do not respond to medical treatment, but there is no evidence supporting the long-term efficacy of this method [13].

Because of these limitations of conventional treatments, herbal medicines are considered as feasible alternatives for the treatment of PD [14]. Herbal medicines have long been used in Eastern countries, but recently these therapies are increasingly being used worldwide [15]. In Korea, many patients who failed to respond to conventional treatments for PD have been treated with herbal medicines. Herbal medicines are relatively well tolerated by patients because of fewer adverse effects and lower recurrence rates associated with them.

During the last few decades, an increasing number of preclinical studies investigating the efficacy of herbal medicines in cell and animal models for PD have been published, but there have not been any review studies on them. This study aims to review the finding of the studies on the* in vitro* and* in vivo* efficacy of herbal medicines for PD. Here, we have summarized the available experimental findings regarding herbal medicines used for PD and their underlying mechanisms.

## 2. Methods

Articles published in English from their inception to April 2014 were searched in the following databases: MEDLINE, EMBASE, and Allied and Complementary Medicine Database (AMED). The search terms were a combination of medical subject heading (MeSH) terms and their synonyms. The search query used was as follows: (herbal medicine (MeSH) OR Chinese herbal drugs (MeSH) OR Chinese traditional medicine (MeSH) OR Korean traditional medicine (MeSH) OR Kampo medicine (MeSH) OR decoction (Title/Abstract) OR tang (Title/Abstract) OR hwan (Title/Abstract)) AND (dysmenorrhea (MeSH) OR primary dysmenorrhea (Title/Abstract)).

**Figure 1:**
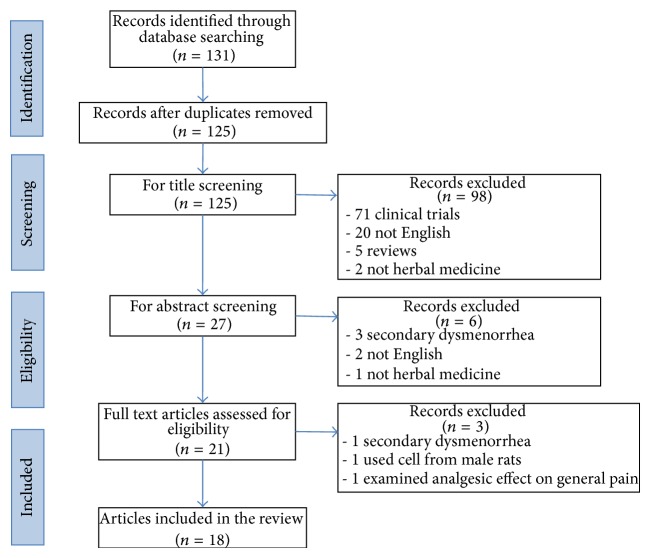
Flow diagram of the study selection process.

All available* in vitro* and* in vivo* studies that assessed the potential effects of herbal medicines on PD were included in our review. Research on the compounds isolated from herbs, individual herbal extracts, or herbal formula decoctions was included. Exclusion criteria were clinical trials of herbal medicines for PD, review articles, or letters.* In vitro* and* in vivo* studies of the compounds or extracts of foods were also excluded. Articles regarding secondary dysmenorrhea or analgesic effects of herbal medicines on general pain were excluded. The titles and abstracts of all the selected articles were examined to eliminate the duplicates. A flow diagram of the article selection process is shown in Figure 1.

## 3. Results

### 3.1. *In Vitro* and* In Vivo* Studies in the Review

In the present review, a total of 18 studies involving herbal medicines exhibited their efficacy on PD. We identified 10* in vitro* studies, five* in vivo* studies, and three studies of both* in vitro* and* in vivo *experiments. The herbal intervention, target cell (or animal model), method of herbal extraction, route of administration, dosage and periods, and outcomes and mechanisms of the* in vitro* and* in vivo* studies are summarized in Tables 1 and 2, respectively.

### 3.2. Herbal Intervention and Frequently Used Herbs

The herbal interventions in the 18 studies comprised three compounds isolated from herbs (three studies), five individual herbal extracts (four studies), and seven herbal formula decoctions (11 studies). Among herbal formula decoctions, Dang-Qui-Shao-Yao-San (Dang-Gui-Shao-Yao-San), Bak Foong pills, Xiang-Fu-Si-Wu decoction, and Shaofu Zhuyu decoction (Shao Fu Zhu Yu decoction) were each used twice (Table 3).* Angelica sinensis* Radix and* Ligusticum chuanxiong* Hort were the most frequently used herbs (nine studies) in herbal formula decoctions (Table 4).

### 3.3. Experimental Models

Experimental target cells and animal models from 18 studies are summarized in Table 5. Uterine muscle from rats or mice was the most frequently used target cells for* in vitro* studies. Uterine muscle contractions include both spontaneous contraction and contraction induced by uterotonic agents. Uterotonic agents used in the studies were oxytocin, PG, acetylcholine, ergonovine, propranolol, KCl, and Ca^2+^. Oxytocin is often used to induce uterine contractions in animals because of its strong constriction promoting effect on uterine arteries [16]. In addition, two types of smooth muscle contractions were examined in the* in vitro* studies: phasic and tonic. Phasic contractions result from a transient increase in cytosolic-free Ca^2+^ concentrations, whereas during tonic contractions the initial peak Ca^2+^ concentration does not return to baseline but reverts to a sustained lower level [17]. Both phasic and tonic contractions cause PD, so both of them were examined in the experiments.

For PD mice model in the* in vivo* studies, estradiol benzoate was often used as a sensitizing agent and uterus contraction was induced by injecting oxytocin [18]. Estradiol benzoate can increase the number of oxytocin receptors in the uterus and result in an increased uterine response to contractile agents [19]. The analgesic activities of herbal medicines were examined by conducting the acetic acid-induced writhing test, oxytocin-induced writhing test, hot-plate test, and formalin-induced licking test.

### 3.4. The Mechanisms of Herbal Medicines for PD

In the majority of the* in vitro* studies (11 out of 13 studies), the inhibitory effects of herbal medicines on uterine contractions were investigated. The mechanisms of herbal medicines for PD are associated with PG level reduction, suppression of cyclooxygenase- (COX-) 2 expression, superoxide dismutase (SOD) activation and malondialdehyde (MDA) reduction, nitric oxide (NO), inducible nitric oxide synthase (iNOS), and nuclear factor-kappa B (NF-*κ*B) reduction, stimulation of somatostatin receptor, intracellular Ca^2+^ reduction, and recovery of phospholipid metabolism (Table 6).

## 4. Discussion

Our review of the literature published from their inception to April 2014 summarized the* in vitro* and* in vivo* studies on the efficacy of herbal medicines for the treatment of PD. Based on the study selection criteria described in Figure 1, we identified 10* in vitro* studies, five* in vivo* studies, and three studies of both* in vitro* and* in vivo* experiments. As a result, a total of three herbal compounds (three studies), five individual herbal extracts (four studies), and seven herbal formula decoctions (11 studies) were found to show inhibitory effects on PD.* Angelica sinensis* Radix and* Ligusticum chuanxiong* Hort were the most frequently used herbs in herbal formula decoctions.

The majority of* in vitro* studies investigated inhibition of uterine contractions. We found that the potential inhibitory activity of herbal medicines could affect different mechanisms of PD. The mechanisms underlying the beneficial effects of herbal medicines on PD are associated with PG level reduction, suppression of COX-2 expression, SOD activation and MDA reduction, NO, iNOS, and NF-*κ*B reduction, stimulation of somatostatin receptor, intracellular Ca^2+^ reduction, and recovery of phospholipid metabolism.

The pathophysiology of PD is due to increased and/or abnormal uterine activity caused by excessive production and release of uterine PG [20]. PD has been reported to lead to increased PG (especially PGE_2_ and PGF_2*α*_) production, which can cause contraction of the blood vessels and myometrium and insufficient blood flow to the endometrium [21]. A previous study revealed that PGE_2_ and PGF_2*α*_ levels in women with PD are higher than those in asymptomatic controls [22]. Rapidly synthesized PG exerts a direct effect on the myometrium, causing the uterine musculature to contract, resulting in constriction of small endometrial blood vessels, tissue ischemia, endometrial disintegration, bleeding, and pain [23]. In this review, shakuyaku-kanzo-to [24], Dang-Gui-Sha-Yao-San (PGF_2*α*_) [25], Bak Foong pills (PGE_2_) [26], and individual and combined extract of* Commiphora myrrha* and* Boswellia carterii* (PGE_2_) [27] were proven to be effective in reducing PG levels.

COX is an enzyme involved in the biosynthesis of PG using arachidonic acid as its principal substrate [28, 29]. The main treatment strategy for the alleviation of PD is the use of NSAIDs, which inhibit COX [30]. The constitutive isoform, COX-1, is expressed in all tissues and most nucleated cells. On the other hand, the inducible form, COX-2, is present only after induction by a variety of factors such as chorionic gonadotropin, cytokines, and tumor promoters [31]. High COX-2 expression leading to increased PG formation during menstruation is the mechanism most likely responsible for PD; this explains the therapeutic efficacy of selective COX-2 inhibitors in ameliorating PD [32]. Several studies have evaluated the effect of COX-2 inhibitors in treating PD [33, 34]. In this review, Dang-Gui-Shao-Yao-San [25], isoliquiritigenin from* Glycyrrhiza glabra* [35], and* Yuanhu* painkillers [36] were proven to effectively suppress COX-2 expression.

Reactive oxygen species have been implicated in the pathogenesis of a variety of injury models. It is possible that PD is one of these conditions. PD has been reported to lead to increase in lipid peroxidation, an index of oxidative stress [37]. MDA is one of the last products of lipid peroxidation, which reflects the degree of lipid peroxidation [38]. Previous studies [39, 40] showed that serum MDA was significantly higher in subjects with PD compared to those in healthy subjects. It is accepted that SOD is one of the most important physiological antioxidants against free radicals and that it prevents subsequent lipid peroxidation [41, 42]. In this review, the main components of* Yuanhu* painkillers [36] decreased the level of MDA and increased the activity of SOD. Antioxidant activity of herbal medicines may play a role in the alleviation of PD.

NO is free radical, and the excessive production of NO is responsible for cytotoxicity by promoting iron-mediated lipid peroxidation and stimulating other proinflammatory enzymes such as COX-2 [43, 44]. Previous studies [40, 45] found that serum NO levels were significantly higher in the patients with PD compared to control group. NO is synthesized by three isoforms of NOS, that is, neuronal NOS (nNOS), endothelial NOS (eNOS), and inducible NOS (iNOS). Although nNOS and eNOS are constitutively expressed, iNOS is expressed in response to interferon-*γ*, lipopolysaccharide, and various inflammatory stimuli [46, 47]. The expression of iNOS is responsible for the production of a significant amount of NO [48]. NF-*κ*B, a small group of closely related transcription factors, is known to play a critical role in coordinating the expression of iNOS and COX-2 [49]. In this review, the main components of* Yuanhu* painkillers [36] reduced iNOS and COX-2 levels and inhibited the subsequent NO in the uterine tissue. They also reduced NF-*κ*B activation, which suggest that their effects on PD may be associated with the reduced iNOS expression level regulated by NF-*κ*B signaling pathway. Isoliquiritigenin from* Glycyrrhiza glabra* [35] and Shao Fu Zhu Yu decoction [50] were also proven to inhibit NO production.

The somatostatin system is also being studied as a possible target for pain control. Somatostatin receptors, which have been implicated in the modulation of nociceptive signals at the level of the spinal cord and are known to be either colocalized or in close proximity to substance P-containing neurons, are differentially regulated during acute and chronic inflammation [51, 52]. In this review, Bak Foong pills [26] stimulated somatostatin receptors, implying that herbal medicines have antinociceptive qualities mediated via the somatostatin pathway.

It is well demonstrated that uterine contraction is associated with external Ca^2+^ influx into myometrial cells. The uterotonic agents that induce uterine contractions increase Ca^2+^ levels via both the influx of extracellular Ca^2+^ through the Ca^2+^ channels and the release of intracellular stored Ca^2+^ [53]. Ca^2+^ signals within the myometrium play an important role in governing uterine excitability and contractility. An increase in Ca^2+^ levels in the uterine smooth muscles induces uterine contraction [54]. Conversely, Ca^2+^ channel blocking agents decrease myometrial contractility and are shown to be beneficial in cases of PD [55]. The studies on Adlay hull extracts [56], Xiang-Fu-Si-Wu decoction [18], and isoliquiritigenin from* Glycyrrhiza glabra* [35] have shown that herbal medicines significantly decreased intracellular Ca^2+^ levels in uterus compared with the controls. One mechanism by which herbal medicines affect PD may involve blocking Ca^2+^ channels to decrease intracellular Ca^2+^ levels.

PD is also associated with endocrinopathy and metabolic abnormality. Recently, disruption of phospholipid metabolism was found to cause PD. Lysophospholipid is the key factor in phospholipid metabolism [57]. The concentrations of these markers were significantly decreased in oxytocin-induced PD rat model. After administration of Xiang-Fu-Si-Wu decoction [58], the concentrations of lysophospholipids were restored to normal levels. This result suggests that perturbations in phospholipid metabolism were associated with PD. Moreover, the therapeutic efficacy of herbal medicine in the animal model may be attributed to its interference with phospholipid.

In the* in vivo* studies, the acetic acid-induced writhing test and the hot-plate test were the main animal models for investigating the analgesic activity of herbal medicines. The acetic acid-induced abdominal writhing test is a visceral and inflammatory pain model. It was reported that PG biosynthesis plays an important role in the nociceptive mechanism in this pain model [59]. In this review, Shao Fu Zhu Yu decoction [50] and isoliquiritigenin from* Glycyrrhiza glabra* [35] produced significant analgesic effects on the number of writhing responses induced by acetic acid, suggesting that they exert peripheral analgesic effects and possible anti-inflammatory activity via inhibition of PG synthesis. The hot-plate test measures the response to an acute noninflammatory nociceptive input and is used to examine centrally acting, but not peripherally acting, analgesic drugs. Isoliquiritigenin from* Glycyrrhiza glabra* [35] effectively reduced acute noninflammatory pain [60], but Shao Fu Zhu Yu decoction [50] did not. Therefore, the inhibitory effect of herbal medicines on acute noninflammatory pain has not been confirmed.

Because the herbal medicines identified in our review include individual herbal extracts and herbal formula decoctions, which have more than a single active component, the observed behaviors may be related to the synergistic actions. An* in vivo* study [27] investigated the anti-inflammatory and analgesic activities of individual and combined extracts from* Commiphora myrrha* and* Boswellia carterii*. The results showed that the combined extracts may be therapeutically more useful for mitigating inflammatory pain than individual herbal extracts. Further, in another study on* Yuanhu* painkillers [36], the synergistic effect of tetrahydropalmatine from* Corydalis yanhusuo* and imperatorin from* Angelica dahurica*, which are the main components of* Yuanhu* painkillers, was significantly better than their individual effects. Tetrahydropalmatine possibly lessens PD by inhibiting the influx of extracellular Ca^2+^, and imperatorin exerts protective effects against PD by abating lipid peroxidation and preventing COX-2 expression. The therapeutic effects of herbal medicines are often the result of comprehensive and integrated outcomes of their active components contained. Thus, combined herbal medicines, acting on diverse factors involved in PD, might provide an alternative approach to treat PD.

## 5. Conclusion

In this review, a variety of herbal medicines exhibited beneficial effects on PD. The major action of herbal medicines is inhibition of uterine contractions. The mechanisms underlying the beneficial effects of herbal medicines on PD are associated with PG level reduction, suppression of COX-2 expression, SOD activation and MDA reduction, NO, iNOS, and NF-*κ*B reduction, stimulation of somatostatin receptor, intracellular Ca^2+^ reduction, and recovery of phospholipid metabolism. Besides, the peripheral analgesic effects and a possible anti-inflammatory activity of herbal medicines were proven in the* in vivo* studies. Herbal medicines are thought to be promising sources in the development of effective therapeutic agents for PD. Further investigations on the appropriate herbal formula and their constituents are recommended.

## Figures and Tables

**Table 1:** *In vitro* studies of herbal medicines for PD.

Study	Herbal medicines	Target cell	Herbal extraction	Dosage	Outcomes and mechanisms
Imai et al. (1995) [24]	Shakuyaku-kanzo-to (TJ-68)	Human endometrium (proliferative-phase from hysterectomy patients due to leiomyoma)	Concentration: 1 to 10 mg/mL Time: 30 min	0, 1, 10, 100, and 1000 *μ*g/mL	(1) PG level↓ (2) Turnover of arachidonic acid in endometrial cells↓

Hsu et al. (2003) [61]	Wen-Jing Tang	Uterine muscle from female Wistar rats (250–350 g)	Extract rate: 35.73%. Solvent: 50% alcohol	0.125–4 mg/mL	Uterine contraction (i) Phasic contraction (induced by PG, acetylcholine, ergonovine, propranolol, and oxytocin)↓ (ii) Tonic contraction (induced by KCl)↓

Hsu et al. (2006) [14]	Dang-Qui-Shao-Yao-San	Uterine muscle from female Wistar rats (250–350 g, 6-7 weeks old)	Extract rate: 30.63%. Solvent: 50% alcohol	0.125–4 mg/mL	Uterine contraction (i) Phasic contraction (induced by PG, acetylcholine, ergonovine, propranolol, and oxytocin)↓ (ii) Tonic contraction (induced by KCl)↓

Du et al. (2006) [62]	Ligustilide (from *Angelica sinensis*)	Uterine muscle from female Wistar rats (180–200 g) and female ICR mice (20–24 g)	Purity: >97%	2–8 *μ*g/mL	Uterine contraction (i) Spontaneous↓ (ii) Induced by PGF_2*α*_, acetylcholine, K^+^, and Ca^2+^-free solution↓

Hua et al. (2008) [25]	Dang-Gui-Shao-Yao-San	Endometrium from nonpregnant female Sprague-Dawley rats (190–210 g)	Solvent: 10 L of 50% ethanol	1, 10, and 100 *μ*g/mL	(1) PGF_2*α*_ level↓ (2) COX-2 mRNA transcription, protein expression, and enzyme activity↓

Hsia et al. (2008) [56]	Adlay hull	Uterine muscle from female Sprague-Dawley rats (200–300 g)	Solvent: 1 L of methanol	0, 25, 75, 175, 375, and 500 *μ*g/mL	(1) Uterine contraction (induced by PGF_2*α*_)↓ (2) Intracellular Ca^2+^↓

Perez-Hernandez et al. (2008) [63]	*Lepechinia caulescens *	Uterine rings from virgin female Wistar rats (240–300 g)	Solvent: 3 L of hexanes	10, 30, and 100 *μ*g/mL	Uterine contraction (induced by KCl)↓

Rowlands et al. (2009) [64]	Bak Foong Pills	Uterine muscle from mature female ICR mice (8–10 weeks old)	Solvent: ethanol	−4.5–2.5 log mg/mL	Uterine contraction (induced by oxytocin)↓

Su et al. (2010) [65]	Shaofu Zhuyu decoction	Uterine muscle from nonpregnant sexually mature female Kunming strain mice (18–22 g, 6-7 weeks old)	Solvent: 5 mL of methanol	6.25–200 *μ*g/mL	Uterine contraction (induced by oxytocin)↓

Liu et al. (2011) [18]	Xiang-Fu-Si-Wu decoction	Uterine muscle from virgin female Wistar rats (180–220 g) and female ICR mice (20–25 g)	Solvent: ethanol (10–80%)	0.01 or 0.001 mg/mL	Uterine contraction (induced by oxytocin)↓

Shi et al. (2012) [35]	Isoliquiritigenin (from *Glycyrrhiza glabra*)	Uterine muscle from nonpregnant female ICR mice (18–22 g)	Purity: >99%	0.5–1000 *μ*M	(1) Uterine contraction (i) Spontaneous↓ (ii) Induced by acetylcholine, KCl, and oxytocin↓ (2) Ca^2+^↓ (3) iNOS↓ (4) COX-1/COX-2↓

Shih and Yang(2012) [66]	Wogonin (from *Scutellaria baicalensis*)	Uterine muscle from nonpregnant female Wistar rats (250–350 g)	Solvent: 50% aqueous ethanol Purity: >99.0%	1–100 *μ*M	Uterine contraction (i) Spontaneous↓ (ii) Induced by oxytocin, PGF_2*α*_, and acetylcholine↓

Jia et al. (2013) [67]	Core licorice extract (CLE)	Uterine muscle from healthy and young adult female ICR mice (25–30 g)	Concentration: 0.5 g/mL	(1) Control group: distilled water (2) CLE group: 0.025, 0.05, and 0.1 mg/mL (3) Reference drugs and CLE group	Uterine contraction (i) Spontaneous↓ (ii) Induced by KCl, acetylcholine, carbachol, oxytocin, and bradykinin↓

**Table 2:** *In vivo* studies of herbal medicines for PD.

Study	Herbal medicines	Animal model	Herbal extraction	Route of administration	Dosage and periods	Outcomes and mechanisms
Hsia et al. (2008) [56]	Adlay hull	Female Sprague-Dawley rats at estrus stage (200–300 g)	Solvent: 1 L of methanol	Subcutaneous injection	5 or 10 mg/kg	Uterine contraction (induced by PGF_2*α*_)↓

Ma et al. (2011) [50]	Shao Fu Zhu Yu decoction	Female ICR mice (18–22 g)	Solvent: 1 L of boiling water Time: twice for 1 h Purity: 80% (by ethanol)	Oral	0.92, 1.84, and 3.68 g/kg	(1) Acetic acid-induced writhing↓ (2) Hot-plate test latency (—) (3) Formalin-induced licking↓ (4) Oxytocin-induced writhing↓ (5) PGE_2_↓ (6) NO↓

Liu et al. (2011) [18]	Xiang-Fu-Si-Wu decoction	Virgin female Wistar rats (180–220 g) and female ICR mice (20–25 g)	Solvent: ethanol (10–80%)	Oral	54.60, 27.30, and 5.46 mg crude herbs/g/d	Ca^2+^ ↓

Shi et al. (2012) [35]	Isoliquiritigenin (from *Glycyrrhiza glabra*)	Nonpregnant ICR mice (18–22 g)	Purity: >99%	Oral	20, 40, and 80 mg/kg	(1) Acetic acid-induced writhing↓ (2) Hot-plate test latency↑

Rowlands et al. (2012) [26]	Bak Foong Pills	Specified pathogen-free C57/B6 mice	—	Oral	0.25, 0.5, 1, and 5 g/kg/day For 3 days	(1) PGE_2_ level↓ (2) Acetic acid-induced writhing↓ (3) Somatostatin receptors 4 and 2 mRNA↓

Su et al. (2012) [27]	(1)* Commiphora myrrha *(MWE) (2)* Boswellia carterii *(FWE) (3) Combined extracts (CWE)	ICR mice (18–22 g)	Solvent: 20 L of water Time: twice for 1 h	Intragastric	(1) Control (2) Dolantin (25 mg/kg) (3) MWE (3.9 g/kg) (4) FEW (6.8 g/kg) (5) CWE (5.2 g/kg) For 3 days	(1) Paw edema (induced by formalin, carrageenan)↓ (2) PGE_2_ level↓ (3) Oxytocin-induced writhing↓

Chen et al. (2013) [36]	*Yuanhu* painkillers (YHP)	Virgin female Wistar rats (250–300 g)	—	Oral	(1) YHP (0.698 g/kg) (2) Tetrahydropalmatine (0.07 g/kg) (3) Imperatorin (0.02 g/kg) (4) Polypharmacy (0.02 g/kg) (5) Tetrahydropalmatine + imperatorin (0.07 g/kg) For 10 days	(1) SOD↑ (2) MDA↓ (3) NO, iNOS↓ (4) i-*κ*B↑ (5) NF-*κ*B↓ (6) COX-2↓

Liu et al. (2014) [58]	Xiang-Fu-Si-Wu decoction	Female Sprague-Dawley rats (220–250 g)	Solvent: 80% ethanol Time: twice for 2 h	Oral	3.78 g crude herbs/kg/d For 7 days	(1) PG level (—) (2) Lysophospholipids↑

**Table 3:** Herbal interventions used in 18 studies.

Herbal intervention	Reference
Herbal compounds	
Isoliquiritigenin (from *Glycyrrhiza glabra*)	[35]
Wogonin (from *Scutellaria baicalensis *Georgi)	[66]
Ligustilide (from *Angelica sinensis*)	[62]
Individual herbal extracts	
Core licorice	[67]
Adlay hull	[56]
*Lepechinia caulescens *	[63]
*Commiphora myrrha *	[27]
*Boswellia carterii *	[27]
Herbal formula decoctions	
Dang-Gui-Shao-Yao-San (Danggui-Shaoyao-San)	[14, 25]
Bak Foong pills	[26, 64]
Xiang-Fu-Si-Wu decoction	[18, 58]
Shaofu Zhuyu decoction (Shao Fu Zhu Yu decoction)	[50, 65]
Shakuyaku-kanzo-to	[24]
Wen-Jing Tang	[61]
*Yuanhu* painkillers	[36]

**Table 4:** Frequently used herbs in herbal formula decoctions.

Herbs	Reference
*Angelica sinensis* Radix *Ligusticum chuanxiong* Hort	Nine studies [14, 18, 25, 26, 50, 58, 61, 64, 65] Nine studies [14, 18, 25, 26, 50, 58, 61, 64, 65]

*Paeonialactiflora *	Eight studies [14, 24–26, 50, 61, 64, 65]

*Corydalis* rhizome	Seven studies [18, 26, 36, 50, 58, 64, 65]

*Atractylodes* rhizome	Six studies [14, 18, 25, 26, 58, 64]

*Cyperi* rhizome *Trogopterori* feces *Cinnamomum* cortex *Poria cocos *	Four studies [18, 26, 58, 64] Four studies [26, 50, 64, 65] Four studies [26, 50, 64, 65] Four studies [14, 25, 26, 64]

*Panax ginseng* CA Meyer *Ophiopogon japonicus Zingiber officinale roscoe *	Three studies [26, 61, 64] Three studies [26, 61, 64] Three studies [50, 61, 65]

*Glycyrrhizae* Radix	Two studies [24, 61]
Rhizoma *Alismatis *	Two studies [14, 25]
Radix *Astragali *	Two studies [26, 64]
*Curcuma aeruginosa *	Two studies [26, 64]
*Ligustrum lucidum *	Two studies [26, 64]
*Phellodendron amurense *	Two studies [26, 64]
*Scutellaria baicalensis *	Two studies [26, 64]
*Polygala tenuifolia *	Two studies [26, 64]
*Eucommia ulmoides *	Two studies [26, 64]
*Linum usitatissimum *	Two studies [26, 64]
*Magnolia officinalis *	Two studies [26, 64]
*Leonurus japonicus *	Two studies [26, 64]
*Lycopus lucidus *	Two studies [26, 64]
*Artemisia argyi *	Two studies [26, 64]
*Amygdalus persica *	Two studies [26, 64]
*Amomumvillosum *	Two studies [26, 64]
*Cornucervi pantotrichum *	Two studies [26, 64]
Fructus*Foeniculum *	Two studies [50, 65]
*Resin commiphora *	Two studies [50, 65]
Pollen*Typhae *	Two studies [50, 65]
Radix *Rehmanniae *Preparata	Two studies [18, 58]
Radix *Aucklandiae *	Two studies [18, 58]

*Pinellia ternata * Breitenbach	One study [61]
*Asini Gelatinum *	One study [61]
*Cinnamomum cassia* Blume	One study [61]
*Paeonia suffruticosa *	One study [61]
*Evodia rutaecarpa *Bentham	One study [61]
*Angelica dahurica *	One study [36]

**Table 5:** Target cells and animal models.

Target cells (*in vitro*)	Reference
Human endometrium	[24]
Uterine muscle from rats or mice	[14, 18, 35, 56, 61–67]
Endometrium from rats	[25]

Animal models (*in vivo*)	Reference

Estrus stage	[56]
Induced by oxytocin following estradiol benzoate	[18, 27, 36, 50, 58]
Induced by acetic acid	[26, 35, 50]

**Table 6:** The mechanisms of herbal medicines for PD.

Main outcomes	Reference
Inhibition of uterine contraction	[14, 18, 35, 56, 61–67]
PG level reduction	[24–27]
Suppression of COX-2 expression	[25, 35, 36]
SOD activation and MDA reduction	[36]
NO, iNOS, and NF-*κ*B reduction	[35, 36, 50]
Stimulation of somatostatin receptor	[26]
Intracellular Ca^2+^ reduction	[18, 35, 56]
Recovery of phospholipid metabolism	[58]

## Abbreviations

COX: Cyclooxygenase
MDA: Malondialdehyde
NF-*κ*B: Nuclear factor-kappa B
NO: Nitric oxide
NOS: Nitric oxide synthase
NSAIDs: Nonsteroidal anti-inflammatory drugs
OCPs: Oral contraceptive pills
PD: Primary dysmenorrhea
PG: Prostaglandin
SOD: Superoxide dismutase.
